# Peritoneal Cancer Index Dominates Prognosis After CRS–HIPEC for Colorectal Peritoneal Metastases: A Consecutive Single-Centre Cohort with 3-Year Follow-Up

**DOI:** 10.3390/cancers17223614

**Published:** 2025-11-10

**Authors:** Michał Kazanowski, Paweł Lesiak, Jędrzej Wierzbicki, Bartosz Kapturkiewicz, Paweł Maciejewski, Marcin Pawłowski, Tomasz Jastrzębski, Marek Bębenek

**Affiliations:** 1 Department of Surgical Oncology, Pulmonology and Hematology, Lower Silesian Center of Oncology, 53-413 Wroclaw, Poland; pawel.lesiak@dcopih.pl (P.L.); jedrzej.wierzbicki@dcopih.pl (J.W.); bartosz.kapturkiewicz@dcopih.pl (B.K.); pawel.maciejewski@dcopih.pl (P.M.); marcin.pawlowski@dcopih.pl (M.P.); marek.bebenek@dcopih.pl (M.B.); 2 Laboratory of Immunopathology, Department of Experimental Therapy, Hirszfeld Institute of Immunology & Experimental Therapy, Polish Academy of Sciences, 53-114 Wroclaw, Poland; 3 Oncological Surgery Department, PCZ Brzeziny Hospital, 95-060 Brzeziny, Poland; jasek@post.pl; 4 Clinic of Gynecology and Obstetrics, Medical University of Gdansk, 80-214 Gdansk, Poland; 5 Faculty of Medicine, Wroclaw University of Science and Technology, 50-370 Wroclaw, Poland

**Keywords:** colorectal cancer, peritoneal metastases, cytoreductive surgery, hyperthermic intraperitoneal chemotherapy, peritoneal cancer index, survival analysis

## Abstract

**Simple Summary:**

Peritoneal metastases from colorectal cancer represent a distinct and aggressive disease pattern with historically poor outcomes. Cytoreductive surgery combined with hyperthermic intraperitoneal chemotherapy (HIPEC) offers selected patients the potential for long-term survival, yet prognostic factors that guide treatment selection remain under investigation. This study evaluated 75 consecutive patients treated at a tertiary oncology centre to identify variables influencing three-year survival after surgery and HIPEC. We found that the Peritoneal Cancer Index, reflecting the extent of peritoneal disease, was the only independent predictor of outcome. Completeness of cytoreduction, operative extent, and choice of intraperitoneal drug showed no additional prognostic effect. These findings support the dominant role of tumour burden in patient selection and underscore the need to integrate molecular and biological markers into future prognostic models.

**Abstract:**

Background: Cytoreductive surgery (CRS) with hyperthermic intraperitoneal chemotherapy (HIPEC) can cure selected patients with colorectal peritoneal metastases (CPM). Real-world prognostic data, especially for the Peritoneal Cancer Index (PCI) and completeness of cytoreduction (CCR), are limited. Methods: We retrospectively analysed 75 consecutive patients treated with CRS + HIPEC at a tertiary centre (2014–2022), giving ≥36 months potential follow-up. Overall survival (OS) was assessed by Kaplan–Meier and Cox models. PCI was grouped 0–10, 11–20, >20; CCR was dichotomised (CCR-0 vs. CCR 1/2). Multivariable analysis included PCI, CCR, and resection extent; HIPEC drug was examined univariately. Results: The median follow-up was 41 months. Crude 3-year OS was 50.7% (38/75). Survival decreased with higher PCI: 69% for 0–10 (*n* = 42), 38% for 11–20 (*n* = 21), and 0% for > 20 (*n* = 4). Versus PCI 0–10, the adjusted hazard ratios (HR) were 3.02 (95% CI 1.52–6.03) for PCI 11–20 and 7.29 (1.72–30.81) for > 20. CCR-0 improved OS univariately (HR 0.43) but was non-significant after adjustment (HR 0.89). Resection limited to the peritoneum (HR 0.99) and choice of intraperitoneal drug showed no independent effect. Conclusions: In this real-world cohort, PCI was the only independent predictor of 3-year survival after CRS + HIPEC for CPM; neither CCR status, surgical extent, nor HIPEC agent altered prognosis once PCI was considered. PCI should therefore remain the principal selection criterion while molecular and biological markers are integrated into future risk models.

## 1. Introduction

Colorectal cancer (CRC) remains one of the most prevalent and lethal malignancies worldwide. At initial presentation 25–35% of patients have stage IV disease and up to 50–60% will develop distant metastases during the course of illness [1]. Peritoneal metastases (PM) constitute a distinct clinical entity, occurring in roughly 10–15% of metastatic CRC cases and carrying the worst prognosis of all metastatic sites. With systemic chemotherapy alone, median overall survival (OS) is typically 12–18 months and the 5-year survival rate remains below 5% in the absence of surgery [2,3].

Over the past two decades, a combined approach using cytoreductive surgery (CRS) and hyperthermic intraperitoneal chemotherapy (HIPEC) has emerged as a potentially curative option for carefully selected patients with limited peritoneal disease. CRS entails complete removal of all macroscopic tumour deposits, while HIPEC delivers heated chemotherapy directly into the peritoneal cavity to eradicate residual microscopic disease. In a pivotal randomised trial, Verwaal et al. demonstrated that CRS + HIPEC increased median OS from 12.6 to 22.3 months and pushed 3-year survival above 40% among optimally selected patients [4].

Subsequent prospective and registry studies have confirmed the long-term benefit of this combined approach, reporting median survivals exceeding 30–40 months in modern cohorts [5,6,7].

More recently, the net benefit of HIPEC has been questioned. The PRODIGE-7 trial found no OS advantage for oxaliplatin-based HIPEC added to complete CRS, prompting a reassessment of HIPEC’s role in routine CRC-PM management [8]. Similarly, the COLOPEC study, which tested prophylactic HIPEC after high-risk colon resection, showed no improvement in disease-free survival [9]. The CAIRO-6 trial is still evaluating peri-operative systemic therapy in combination with CRS ± HIPEC and may further redefine best practice [10].

Despite these developments, real-world survival data stratified by two key prognostic variables—the Peritoneal Cancer Index (PCI) and the completeness of cytoreduction (CCR)—remain sparse. Clarifying how PCI and CCR shape outcomes in everyday clinical practice is critical for optimising patient selection, refining surgical decision-making, and ultimately improving survival.

In this study, we analysed 3-year survival in a consecutive, single-centre cohort of patients who underwent CRS + HIPEC for CRC-related peritoneal metastases, with particular emphasis on PCI- and CCR-based stratification.

We aimed to identify independent prognostic factors for overall survival after CRS–HIPEC in colorectal peritoneal metastases, with particular emphasis on the prognostic weight of the Peritoneal Cancer Index.

## 2. Materials and Methods

### 2.1. Study Design and Patient Population

We conducted a retrospective, single-centre cohort study of all patients with histologically proven colorectal adenocarcinoma and peritoneal metastases (PM) who underwent cytoreductive surgery (CRS) with hyperthermic intraperitoneal chemotherapy (HIPEC) at our tertiary surgical oncology unit between January 2014 and December 2024. In total, 115 CRS–HIPEC procedures were performed during this calendar period. For survival analyses, we restricted the dataset to operations carried out on or before 8 May 2022, guaranteeing a minimum potential follow-up of 36 months at the data-cut-off (9 May 2025). This yielded the final analysis cohort of 75 patients.

The eligibility criteria were the following: (i) curative-intent CRS–HIPEC for PM originating from colorectal adenocarcinoma and (ii) complete peri-operative records. The exclusion criteria were as follows: non-colorectal primary tumour, palliative-intent surgery, or incomplete key data. Staging or diagnostic laparoscopy was not routinely performed before CRS–HIPEC during the study period; all patients underwent pre-operative contrast-enhanced CT for disease mapping and PCI estimation.

Candidates for CRS–HIPEC were discussed at a multidisciplinary tumour board. Eligibility required ECOG ≤ 2, adequate organ function, and absence of extraperitoneal disease. CRS–HIPEC was generally not pursued for PCI > 20, although in selected low-grade histologies a complete cytoreduction was attempted if deemed technically feasible.

### 2.2. Data Collection and Variable Definitions

Demographic, surgical, and oncological variables were extracted from the institutional electronic medical record (CliniNet^®^ and Hipokrates^®^) via the MedStreamDesigner^®^ data warehouse and anonymised prior to analysis.

- Pre-operative qualification for CRS–HIPEC was based on contrast-enhanced computed tomography (CT) performed as part of staging work-up, with the Peritoneal Cancer Index (PCI) estimated to assess disease extent and operability. The definitive PCI was recorded intra-operatively during laparotomy using the standard 13-region Sugarbaker method and used for all analyses.
- Completeness of cytoreduction (CCR) was assigned at the end of CRS:
  ○ CCR-0—no macroscopic residual disease
  ○ CCR-1—residual nodules ≤ 2.5 mm
  ○ CCR-2—residual nodules > 2.5 mm
- The HIPEC procedure was performed immediately after CRS using a closed-abdomen technique in all cases. The most commonly used agent was oxaliplatin (460 mg/m^2^), administered over 30 min at 42–43 °C, and used in 70.7% of patients. Other agents included doxorubicin (14.7%), mitomycin C (10.7%), and cisplatin (4.0%), chosen according to institutional protocols based on prior systemic therapy, tumour biology, and known drug intolerances. No intravenous chemotherapy was co-administered. The perfusion parameters were continuously monitored during the procedure. The extent of resection was coded as peritoneum-only versus multivisceral, according to the operative report.

### 2.3. Survival End Points

The overall survival (OS) was calculated from the date of CRS–HIPEC to death from any cause or last known follow-up. For the predefined 3-year OS analysis, patients were categorised as one of the following:

- Survivor—no recorded death within 36 months of surgery (blank follow-up dates were considered alive under this rule);
- Non-survivor—death recorded ≤ 36 months;
- Censored—not applicable, as the study window ensured ≥ 36 months of potential follow-up for every case.

Stratification:

Prognostic analyses used the following groupings:

- PCI: 0–10, 11–20, >20
- CCR: CCR-0 vs. CCR-1/2

Post-operative surveillance followed institutional and regional oncology protocols. Patients residing within the referral area were typically followed at our centre with clinical visits and serum CEA measurements every three months during the first post-operative year, and contrast-enhanced CT every six months for the first three years, then annually thereafter. Patients referred from other regions were usually followed at their local oncology centres, and detailed follow-up imaging data were therefore not consistently available for all cases.

### 2.4. Statistical Analysis

Baseline characteristics are reported as medians with inter-quartile range (IQR) for continuous variables and counts with percentages for categorical variables. Differences between groups were tested with Fisher’s exact test (categorical) or the Mann–Whitney U test (continuous). Survival curves were generated by the Kaplan–Meier method and compared with the log-rank test. Median OS and 3-year survival proportions are presented with 95% confidence intervals (CI). Univariable and multivariable Cox proportional hazards models were built to identify predictors of OS; variables entered a priori were PCI group, CCR status, and extent of resection. Model discrimination was assessed with Harrell’s concordance index and overall fit with the likelihood-ratio test. Eight patients had missing PCI values and five had missing CCR values; these cases were excluded from the relevant subgroup analyses (PCI analyses *n* = 67; CCR analyses *n* = 70). No data imputation was performed.

All analyses were performed using Python version 3.10 (Python Software Foundation, Wilmington, DE, USA) with the pandas and lifelines 0.27 libraries, and cross-checked in Jamovi version 2.4 (The Jamovi Project, Sydney, Australia) and Statistica version 13.3 (TIBCO Software Inc., Palo Alto, CA, USA). A two-sided *p* < 0.05 was considered statistically significant.

### 2.5. Ethical Considerations

The study protocol was approved by the Institutional Ethics Committee. All procedures conformed to the principles of the Declaration of Helsinki and relevant national regulations. Written informed consent for treatment and use of anonymised data for research was obtained from each patient or legal representative.

## 3. Results

### 3.1. Patient Characteristics

Between January 2014 and 8 May 2022, 75 consecutive patients underwent cytoreductive surgery combined with hyperthermic intraperitoneal chemotherapy for colorectal peritoneal metastases and therefore met the predefined eligibility window for the 3-year survival analysis. The median age at operation was 60 years (inter-quartile range [IQR] 54–67), and the cohort comprised 34 men (45%) and 41 women (55%).

A peri-operative Peritoneal Cancer Index (PCI) was documented in 67 (89%) cases; the median PCI for the whole group was 10 (IQR 7–15). Complete macroscopic cytoreduction (CCR-0) was achieved in 58 of 75 patients (77%), while 12 patients (16%) had minimal residual disease (CCR-1/2) and 5 had missing CCR data. In total, 23 procedures (31%) were limited to peritoneum-only stripping, whereas 52 (69%) required additional visceral or parenchymal resections. Oxaliplatin was the intraperitoneal drug of choice in 53 patients (71%); doxorubicin, mitomycin C, and cisplatin were used in 11 (15%), 8 (11%), and 3 (4%) cases, respectively. Baseline characteristics are summarised in Table 1 (below).

### 3.2. Survival by Peritoneal Cancer Index

**Figure 1:**
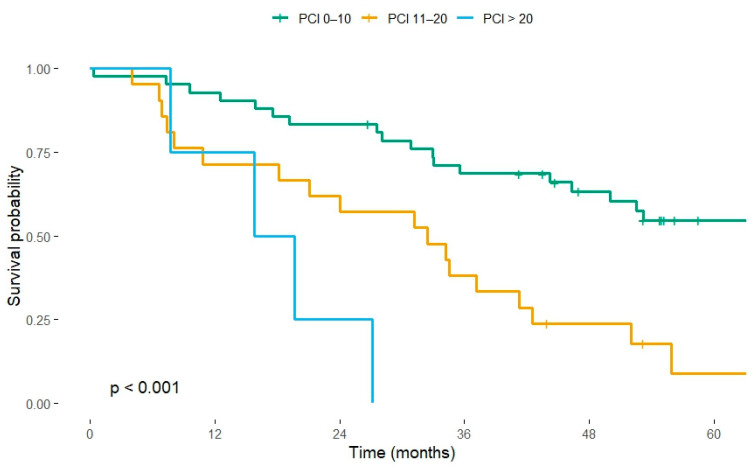
Kaplan–Meier overall-survival curves stratified by Peritoneal Cancer Index (PCI). Group sizes: PCI 0–10 (*n* = 42), PCI 11–20 (*n* = 21), PCI > 20 (*n* = 4). Log-rank test *p* < 0.001. Vertical ticks indicate censored observations.

PCI stratification revealed a clear gradient in outcome (log-rank *p* < 0.001; Figure 1). Among the 42 patients with PCI 0–10, crude 3-year overall survival (OS) was 69% (29/42), and the median OS was not reached at the time of censoring. In contrast, the 21 patients with PCI 11–20 had a 3-year OS of 38% (8/21); their median OS was 986 days (95% confidence interval [CI] 553–1096). All four patients with PCI > 20 died within three years, yielding a median OS of 540 days (95% CI 235–NA) and a 3-year survival of 0%.

On univariable Cox regression (PCI 0–10 as reference), the hazard of death increased three-fold for PCI 11–20 (HR 3.10, 95% CI 1.60–6.00, *p* = 0.001) and eight-fold for PCI > 20 (HR 8.15, 95% CI 2.54–26.18, *p* < 0.001).

### 3.3. Completeness of Cytoreduction

**Figure 2:**
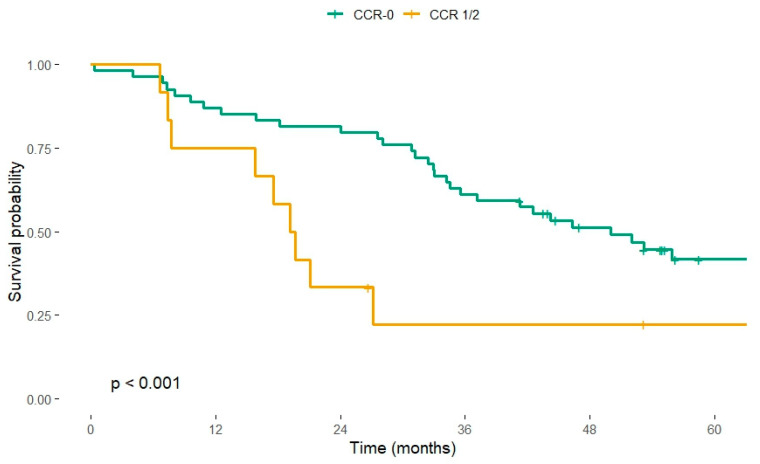
Kaplan–Meier overall-survival curves according to completeness of cytoreduction (CCR). CCR-0 (*n* = 58), CCR 1/2 (*n* = 12). Log-rank test *p* < 0.001. Vertical ticks indicate censored observations.

CCR status also influenced crude survival, although its effect diminished after adjusting for tumour burden. Of the 70 patients with a recorded CCR score, 58 achieved CCR-0 and 12 had CCR-1/2. Median OS was 1 095.8 days (≈ 36.0 months; 95% CI 1050–NA) in the CCR-0 group versus 584 days (≈ 19.2 months; 95% CI 481–1 096) in the CCR-1/2 group. Corresponding 3-year OS proportions were 41.4% (24/58) and 25.0% (3/12), respectively. Univariable analysis confirmed a protective effect of complete cytoreduction (HR 0.43, 95% CI 0.23–0.81, *p* = 0.009). However, when PCI and extent of resection were entered into the multivariable model, CCR-0 lost statistical significance (adjusted HR 0.89, 95% CI 0.35–2.21, *p* = 0.795), indicating that its benefit was largely mediated by tumour burden (Figure 2).

Because only two patients carried a CCR-2 designation, a separate comparison of CCR-2 versus CCR-0/1 was not performed; an exploratory Cox model (HR 0.79, *p* = 0.817) was uninterpretable owing to the very small sample and event count.

### 3.4. Extent of Resection

Patients whose surgery was confined to the peritoneum (*n* = 23) enjoyed a numerically longer median OS—44.2 months (95% CI 27.1–NA)—than those who underwent multivisceral resection (*n* = 52; median OS 35.3 months, 95% CI 31.2–49.9). The respective 3-year OS rates were 43.5% and 32.7%. Nevertheless, neither the univariable comparison (HR 0.74, 95% CI 0.38–1.43, *p* = 0.373) nor the multivariable model (adjusted HR 0.99, *p* = 0.969) demonstrated a statistically significant difference.

### 3.5. Relationship Between PCI and Completeness of Cytoreduction

As expected, higher Peritoneal Cancer Index (PCI) values were associated with a lower likelihood of achieving complete macroscopic cytoreduction. In the entire colorectal CRS–HIPEC cohort (*n* ≈ 111), the frequency of CCR-0 decreased markedly across PCI strata (χ^2^ = 18.7, *p* < 0.001) (Table 2). This exploratory analysis used the full institutional dataset, whereas the 3-year survival analysis described below was restricted to 75 patients with sufficient follow-up potential.

The strong correlation between PCI and cytoreduction completeness underscores the increasing technical difficulty of achieving macroscopic clearance in high-burden disease and supports PCI as the principal pre-operative determinant of feasibility and prognosis.

### 3.6. Influence of the HIPEC Agent

Median OS varied across the four intraperitoneal drugs—37.1 months for oxaliplatin, 49.9 months for doxorubicin, 27.1 months for cisplatin, and 18.5 months for mitomycin C, but none of these pairwise comparisons reached statistical significance in univariable Cox testing (all *p* > 0.30). Crude 3-year OS proportions were 55% (29/53) for oxaliplatin, 45% (5/11) for doxorubicin, 33% (1/3) for cisplatin, and 25% (2/8) for mitomycin C.

### 3.7. Multivariable Cox Model

The final multivariable Cox proportional hazards model incorporated PCI group, CCR status, and extent of resection. PCI remained the sole independent predictor of mortality. Relative to PCI 0–10:

- PCI 11–20 carried an adjusted HR of 3.02 (95% CI 1.52–6.03, *p* = 0.002).
- PCI > 20 carried an adjusted HR of 7.29 (95% CI 1.72–30.81, *p* = 0.007).

Neither CCR-0 (adjusted HR 0.89, *p* = 0.795) nor peritoneum-only resection (adjusted HR 0.99, *p* = 0.969) retained statistical significance. The model demonstrated moderate discrimination with a concordance index of 0.667, and the overall likelihood-ratio test was highly significant (χ^2^ = 19.4, *p* = 0.002).

### 3.8. Post-Operative Morbidity and Early Outcomes

Among all analysed CRS–HIPEC procedures, the overall reoperation rate was 5.7%, mainly for bowel necrosis or post-operative bleeding, while 1.9% of patients required readmission within 30 days, predominantly for wound complications or subileus. The incidence of reoperation increased with higher Peritoneal Cancer Index (PCI 0–10: 2.9%, PCI 11–20: 8.9%, PCI > 20: 8.8%; *p* = 0.07), whereas readmission rates were not significantly associated with PCI (*p* = 0.66).

Taken together, these findings reinforce the dominant prognostic role of tumour burden, measured by the Peritoneal Cancer Index, in patients undergoing CRS-HIPEC for colorectal peritoneal metastases, while completeness of cytoreduction, surgical extent and choice of intraperitoneal drug exert no independent influence once PCI is taken into account.

### 3.9. Predictors of Overall Survival

On univariable analysis, increasing PCI was strongly associated with higher mortality risk (PCI 11–20: HR 3.10; PCI > 20: HR 8.15; both *p* ≤ 0.001), and complete cytoreduction was associated with improved survival (CCR-0 vs. CCR-1/2: HR 0.43; *p* = 0.009). In the multivariable model including PCI group, CCR, and extent of resection, PCI remained the sole independent predictor (PCI 11–20: aHR 3.02, *p* = 0.002; PCI > 20: aHR 7.29, *p* = 0.007), whereas CCR (aHR 0.89, *p* = 0.795) and peritoneum-only resection (aHR 0.99, *p* = 0.969) were not independently associated with survival (Table 3).

## 4. Discussion

The present study reflects the development of a regional CRS–HIPEC referral programme in southwestern Poland, providing one of the largest consecutive national series of colorectal peritoneal metastases treated in a single institution.

Our contemporary, single-centre audit shows that tumour burden, quantified by the Peritoneal Cancer Index (PCI), is the only independent driver of outcome after CRS + HIPEC for colorectal peritoneal metastases. This mirrors recent series, where low PCI and complete cytoreduction were consistently associated with long-term survival after CRS–HIPEC for colorectal peritoneal metastases [11,12]. The crude 3-year overall survival (OS) for the cohort was 50.7%, yet survival separated sharply by PCI: 69% for PCI 0–10, 38% for PCI 11–20, and 0% for PCI > 20. In multivariable analysis, the hazard of death rose three-fold for PCI 11–20 and eight-fold for PCI > 20 compared with PCI 0–10. These findings reaffirm the long-standing observation that a low PCI predicts superior survival because it both reflects limited microscopic disease and permits more reliable complete cytoreduction [13,14]. The data also support the pragmatic—but often debated—upper eligibility threshold of PCI 20 adopted by most high-volume centres [15,16].

CCR-0 conferred a clear benefit in univariable testing (3-year OS 41% vs. 25%; HR 0.43) but lost significance once PCI and operative extent were controlled for (adjusted HR 0.89). This attenuation is expected, because achieving CCR-0 becomes progressively harder as PCI rises. Nevertheless, the principle of striving for macroscopic clearance remains valid and is repeatedly confirmed in multi-institutional series [17,18]. What is increasingly recognised, however, is that macroscopic completeness alone may not translate into a long-term cure when micrometastatic or biologically aggressive disease persists [19,20].

Growing molecular evidence illustrates this point. KRAS, BRAF, and PIK3CA mutations, high microsatellite instability (MSI-H), and the poor-prognosis CMS4 transcriptomic subtype have each been linked to rapid peritoneal spread and resistance to intraperitoneal chemotherapy [21,22]. In a recent multicentre analysis, BRAF-mutated tumours fared poorly after CRS–HIPEC regardless of PCI or CCR status [21]. Incorporating such markers, alongside liquid-biopsy or ctDNA read-outs, may soon refine patient selection beyond anatomical scoring alone [23].

Even as PCI remains indispensable, its use as a rigid cut-off (≤10 or ≤20) can mask prognostic gradients. Continuous PCI models and machine learning (ML) nomograms that blend spatial score, organ involvement, and basic molecular data outperform categorical thresholds in the PSOGI registry and the Netherlands Cancer Institute datasets [24,25]. We anticipate that future risk calculators will combine PCI, molecular subtype, and systemic inflammatory markers into an integrated index.

Neither the intraperitoneal drug nor the extent of resection altered survival in our cohort. Oxaliplatin—used in 71% of cases—showed no advantage over doxorubicin, mitomycin C, or cisplatin, echoing the neutral HIPEC-drug signal in PRODIGE-7 and multiple meta-analyses [8,26,27]. These results complement observations from the major randomised trials evaluating CRS–HIPEC in colorectal peritoneal metastases. In the PRODIGE-7 trial, which compared oxaliplatin-based HIPEC versus CRS alone, PCI was the strongest stratification variable for overall survival, and patients with PCI > 20 were excluded because of poor prognosis. Similarly, in the COLOPEC trial, which tested adjuvant HIPEC after curative resection of T4 or perforated tumours, PCI and completeness of cytoreduction were key determinants of recurrence risk, but PCI was not formally incorporated into patient selection. Our findings reinforce that PCI, rather than the HIPEC regimen itself, remains the principal driver of outcome after CRS–HIPEC, emphasising the need for accurate pre-operative PCI assessment and consistent reporting in future trials.

Similarly, multivisceral resections did not improve OS relative to peritoneum-only stripping once PCI was accounted for (adjusted HR 0.99). These findings argue for a restrained operative approach guided by oncological rationale rather than by the pursuit of ever-wider resections [28,29].

Diagnostic laparoscopy with PCI assessment and targeted biopsies may further refine pre-operative stratification and facilitate integration of molecular profiling into treatment sequencing. Incorporating minimally invasive staging and comprehensive genomic characterisation could help personalise therapeutic strategies for colorectal peritoneal metastases [30].

Comparable outcomes have been reported in large multi-institutional datasets from France (PSOGI/RENAPE) and Japan, where PCI consistently emerged as the dominant prognostic factor [31,32]. These cross-regional similarities suggest that the prognostic impact of PCI is largely independent of institutional volume or chemotherapeutic regimen, reinforcing its global validity as a patient-selection tool.

## 5. Limitations

This study is retrospective and single centre, exposing it to selection bias and limiting external generalisability. Although every patient had ≥36 months potential follow-up, 27 survivors lacked a documented clinic visit beyond three years, so crude 3-year OS may slightly over- or under-estimate Kaplan–Meier survival. The sample size was modest, leaving the CCR-2 subgroup (*n* = 2) and the smaller HIPEC-drug strata under-powered. Finally, molecular data were not available, precluding assessment of genotype–phenotype interactions.

Molecular analysis of KRAS, BRAF, and PIK3CA mutations, high microsatellite instability (MSI-H), and the poor-prognosis CMS4 transcriptomic subtype have each been linked to rapid peritoneal spread and resistance to intraperitoneal chemotherapy [33]. These molecular parameters were not available in our retrospective dataset but should be integrated into future prognostic models alongside PCI to improve patient selection.

## 6. Conclusions

In our real-world series, PCI eclipsed all other clinicopathological variables as a determinant of survival after CRS + HIPEC for colorectal peritoneal metastases. Until robust molecular stratifiers are validated, PCI should remain the primary gate-keeping criterion, with CCR-0 pursued where feasible. Ongoing trials, such as CAIRO-6 and DRAGON, will clarify the role of peri-operative systemic therapy, immunomodulation, and agent-specific HIPEC. Future prognostic frameworks should integrate continuous PCI, molecular subtype, and circulating-tumour biomarkers to guide truly personalised cytoreductive strategies.

## Figures and Tables

**Table 1:** Patient demographics and baseline characteristics (*n* = 75).

Variable	Value
Total patients	75
Median age (IQR)	60 (54–67)
Gender (male/female)	34/41
Median PCI (IQR)	10 (6–15)
CCR-0 (%)	58 (77.3%)
Peritoneum only resection (%)	23 (30.7%)
Most common HIPEC agent (oxaliplatin) (%)	53 (70.7%)

Note: Table values are based on available data. Some variables contain missing values, so subgroup counts may be <75.

**Table 2:** Relationship between Peritoneal Cancer Index (PCI) and completeness of cytoreduction (CCR) in the full colorectal CRS–HIPEC cohort.

PCI Group	CCR-0	CCR-1/2	CCR-0 (%)
0–10	55	5	92%
11–20	36	8	82%
>20	1	6	14%

**Table 3:** Predictors of overall survival after CRS–HIPEC for colorectal peritoneal metastases (Cox regression; *n* = 75).

Variable	Category (Reference)	Univariable HR (95% CI)	*p*	Multivariable HR (95% CI)	*p*
Peritoneal Cancer Index (PCI)	0–10 (ref)	—	—	—	—
	11–20	3.10 (1.60–6.00)	0.001	3.02 (1.52–6.03)	0.002
	>20	8.15 (2.54–26.18)	<0.001	7.29 (1.72–30.81)	0.007
Completeness of cytoreduction (CCR)	CCR-0 (ref)	—	—	—	—
	CCR 1/2	2.33 (1.23–4.35) †	0.009	1.12 (0.45–2.86) ‡	0.795
Extent of resection	Multivisceral (ref)	—	—	—	—
	Peritoneum only	0.74 (0.38–1.43)	0.373	0.99 (0.48–2.04)	0.969

Note: Hazard ratios (HR) < 1 favour improved survival. Multivariable model included PCI group, CCR status, and extent of resection. Model performance: Harrell’s C-index = 0.667; Likelihood-ratio test χ^2^ = 19.4, *p* = 0.002. † Inverse of CCR-0 HR 0.43 (0.23–0.81). ‡ Inverse of CCR-0 aHR 0.89 (0.35–2.21). PCI grouped as 0–10, 11–20, >20; CCR dichotomized (CCR-0 vs. CCR-1/2). “Peritoneum only” denotes no multivisceral resection. HIPEC agent was explored univariately and showed no significant association; it was not entered into the multivariable model to avoid overfitting.

## Data Availability

The data that support the findings of this study are derived from the institutional clinical database of the Lower Silesian Center of Oncology, Pulmonology and Hematology, Wroclaw, Poland. Due to privacy and ethical restrictions, the data are not publicly available but can be obtained from the corresponding author upon reasonable request and with permission of the institutional ethics committee.
